# Effect of EPA on Neonatal Pig Sertoli Cells “*In Vitro*”: A Possible Treatment to Help Maintain Fertility in Pre-Pubertal Boys Undergoing Treatment With Gonado-Toxic Therapies

**DOI:** 10.3389/fendo.2021.694796

**Published:** 2021-05-20

**Authors:** Iva Arato, Veronica Ceccarelli, Francesca Mancuso, Catia Bellucci, Cinzia Lilli, Piero Ferolla, Katia Perruccio, Alessandro D’Arpino, Maria Chiara Aglietti, Riccardo Calafiore, Don F. Cameron, Mario Calvitti, Tiziano Baroni, Alba Vecchini, Giovanni Luca

**Affiliations:** ^1^ Department of Medicine and Surgery, University of Perugia, Perugia, Italy; ^2^ Department of Medical Oncology, Multidisciplinary Neuroendocrine Tumours (NET) Group, Umbria Regional Cancer Network and University of Perugia, Perugia, Italy; ^3^ Pediatric Oncology Hematology, Department of Mother and Child Health, Perugia, Italy; ^4^ Pharmacy Unit, Santa Maria della Misericordia Hospital, Perugia, Italy; ^5^ International Biotechnological Center for Endocrine, Metabolic and Embryo-Reproductive Translational Research (CIRTEMER), Department of Medicine and Surgery, University of Perugia, Perugia, Italy; ^6^ Morisani College of Medicine FL, University of South Florida, Tampa, FL, United States; ^7^ Division of Medical Andrology and Endocrinology of Reproduction, Saint Mary Hospital, Terni, Italy

**Keywords:** eicosapentaenoic acid, Sertoli cells, infertility, chemotherapeutic agents, pre-pubertal boys

## Abstract

The incidence of cancer in pre-pubertal boys has significantly increased and, it has been recognized that the gonado-toxic effect of the cancer treatments may lead to infertility. Here, we have evaluated the effects on porcine neonatal Sertoli cells (SCs) of three commonly used chemotherapy drugs; cisplatin, 4-Hydroperoxycyclophosphamide and doxorubicin. All three drugs induced a statistical reduction of 5-hydroxymethylcytosine in comparison with the control group, performed by Immunofluorescence Analysis. The gene and protein expression levels of GDNF, were significantly down-regulated after treatment to all three chemotherapy drugs comparison with the control group. Specifically, differences in the mRNA levels of GDNF were: 0,8200 ± 0,0440, 0,6400 ± 0,0140, 0,4400 ± 0,0130 fold change at 0.33, 1.66, and 3.33μM cisplatin concentrations, respectively (**p < 0.01 at 0.33 and 1.66 μM *vs* SCs and ***p < 0.001 at 3.33μM *vs* SCs); 0,6000 ± 0,0340, 0,4200 ± 0,0130 fold change at 50 and 100 μM of 4-Hydroperoxycyclophosphamide concentrations, respectively (**p < 0.01 at both these concentrations *vs* SCs); 0,7000 ± 0,0340, 0,6200 ± 0,0240, 0,4000 ± 0,0230 fold change at 0.1, 0.2 and 1 µM doxorubicin concentrations, respectively (**p < 0.01 at 0.1 and 0.2 μM *vs* SCs and ***p < 0.001 at 1 μM *vs* SCs). Differences in the protein expression levels of GDNF were: 0,7400 ± 0,0340, 0,2000 ± 0,0240, 0,0400 ± 0,0230 A.U. at 0.33, 1.66, and 3.33μM cisplatin concentrations, respectively (**p < 0.01 at both these concentrations *vs* SCs); 0,7300 ± 0,0340, 0,4000 ± 0,0130 A.U. at 50 and 100 μM of 4- Hydroperoxycyclophosphamide concentrations, respectively (**p < 0.01 at both these concentrations *vs* SCs); 0,6200 ± 0,0340, 0,4000 ± 0,0240, 0,3800 ± 0,0230 A.U. at 0.l, 0.2 and 1 µM doxorubicin concentrations, respectively (**p < 0.01 at 0.1 and 0.2 μM *vs* SCs and ***p < 0.001 at 1 μM *vs* SCs). Furthermore, we have demonstrated the protective effect of eicosapentaenoic acid on SCs only at the highest concentration of cisplatin, resulting in an increase in both gene and protein expression levels of GDNF (1,3400 ± 0,0280 fold change; **p < 0.01 *vs* SCs); and of AMH and inhibin B that were significantly recovered with values comparable to the control group. Results from this study, offers the opportunity to develop future therapeutic strategies for male fertility management, especially in pre-pubertal boys.

## Introduction

Pre-pubertal boy’s cancer survival rate has markedly increased in recent decades, with the current 5-year survival rate across all childhood cancers above 80% (1), for both early diagnosis and improved cancer treatment protocols (2). At present, alkylating and alkylating-like agents such as cyclophosphamide and cisplatin, as well as doxorubicin, are three drugs commonly used in treatment regimens for a wide range of childhood cancer, resulting in more clinical information available, including that of patient serum concentrations. The alkylating agents are considered to be highly toxic agents intercalating into DNA, disrupting basic cellular processes, with the doxorubicin that is often considered to be the least toxic of the agents (3). It has long been recognized that chemotherapy treatment can have adverse effects on male reproduction, including impairment of subsequent fertility. In fact, it is estimated that nearly half of male pre-pubertal cancer survivors will experience difficulties to conceive a child during adulthood presenting, therefore, a significant quality-of-life issue for many men (4–6).

The future fertility in men has been extensively studied and there are now clear links between the use of chemotherapy drugs in treatment regimens for childhood cancers and subsequent impairment of their fertility status (7, 8). Indeed, a study by Chow et al., 2016, has shown a reduced fertility rate for male and, to a lesser extent, female survivors of childhood cancer in comparison to their siblings. Several studies indicate that chemotherapy treatments in pre-pubertal subjects, are able to reduce the overall size of the testis where there is depletion of the germ cells (spermatogonia Ad and Ap), resulting in Sertoli cell-only tubules, as well as interstitial fibrosis and basement membrane thickening (9–11). It is also known that treatment with chemotherapeutic drugs induces a reduction of both Sertoli Cell (SCs) viability (12) and function (13) resulting in a complete inhibition of spermatogenesis and, finally, in sterility due to azoospermia (7, 8).

The pre-pubertal testis appears to be more sensitive to chemotherapy treatments than the adult testis because the testicular environment is not quiescent but rather is in a constant state of turnover of early germ cells (14, 15). Establishment of spermatogenesis during puberty will depend on the degree of damage caused by the treatment either directly to spermatogonial stem cells (GSC) or indirectly by impairment of Sertoli and Leydig cells. Complete depletion of GSC results in permanent azoospermia (8). Therefore, the issue of infertility treatment to maintain the ability to genetically father one’s own children is a major concern for those young survivors who were treated with gonado-toxic agents (16–18).

Preservation of semen before cancer treatment is currently the only method of preserving future male fertility potential. Obviously, this technique is not an option for pre-pubertal patients who do not yet produce mature spermatozoa that can be used for routine sperm cryopreservation (19–21). Recently, the same European center has started to cryopreserve immature testicular tissue from pre-pubertal boys before the commencement of chemotherapy treatment. However, it is not yet certain if such cryopreserved tissue can be successfully used later to restore fertility in humans, as production of viable sperm from such tissue has yet to be shown (2, 16).

The SCs, the only somatic cell type in the seminiferous tubules, can be considered the real “director” of spermatogenesis (22). They directly interact with germ cells, by the secretion of specific factors, to control their proliferation and differentiation toward spermatogenic completion (23–27). SCs growth factors, such as glial cell line-derived neurotrophic factor (GDNF), have been identified as the most important upstream factors that regulate SSC germ cell self-renewal and spermatocyte meiosis (28). In particular, the GDNF is a member of the transforming growth factor beta (TGF-β) superfamily that binds to the RET/GFRA1 receptor complex at the surface of undifferentiated spermatogonia, and is known for its ability to drive GSC self-renewal and proliferation of their direct cell progeny (29, 30).

The importance of GDNF for germ cell development was uncovered by the seminal work of Meng and colleagues (31) who demonstrated that mice heterozygous for GDNF, though fertile, exhibit increased numbers of seminiferous tubules lacking spermatogonia as the animals aged. Conversely, transgenic animals overexpressing GDNF display an accumulation of undifferentiated spermatogonia. Thereafter, it was demonstrated that GFRA1 and RET proteins and mRNA are expressed in these cells (32–34), confirming that they are able to respond to GDNF influence.

In the last few years evidence has been found linking germ cell toxicity mediated by chemotherapy drugs to the appearance of epigenetic modifications in treated subjects (35, 36).

DNA methylation, considered as one of the main epigenetic mechanisms, is already known to influence male fertility (37). In particular, DNA methylation converts cytosine to 5’-methyl cytosine by at least 5 types of DNA methyl-transferases (DNMTs) (38). Such methylation occurs in the so-called “CpG islands” (cytosine-phosphate-guanine), which are regions of DNA rich in dinucleotide formed by cytosine that precedes guanine. This mechanism, together with the eventual deacetylation of histone lysine, is responsible for the compact conformation of chromatin which inactivates the transcription of the genes of interest by preventing the access of transcription factors to promoter regions rich in CpG islands (39). Gene silencing by DNA hypermethylation is the final result of this process (40). DNA methylation and histone modifications are important regulators involved in chromatin remodeling essential for the transcription of several genes in the testes, indicating a direct influence of epigenetic mechanisms on the process of spermatogenesis. On the contrary, an aberrant methylation of genomic DNA, which affects about 14% of the paternal genes, is associated with oligospermia or oligo-astenoterazoospermia (41). Moreover, recent data show that GDNF expression is regulated by epigenetic mechanisms in glioma and Sertoli cells (42, 43).

It was demonstrated that eicosapentaenoic acid (EPA), a fatty acid with anti-cancer properties, is able to decrease DNA methylation levels through the activation of ten-eleven translocation enzymes proteins (TETs). They convert 5-methylcytosine (5mC) to 5-hydroxymethylcytosine (5hmC), thus promoting re-expression of silenced genes by hypermethylation (44).

Furthermore, EPA can inhibit histone deacetylase 1 (HDAC1) and DNMT expression and activity, which are the enzymes responsible, respectively, for the deacetylation of histone lysine residues and for the methylation of cytosine in the CpG islands of DNA (45). Previous studies demonstrated the protective effect of EPA through the inhibition of apoptosis, lipoperoxidation and the production of oxygen-active radical species in mouse SCs cultures (46).

In this study, we report the effects on porcine neonatal SCs “*in vitro*” of three different chemotherapeutic agents, cisplatin, 4-Hydroperoxycyclophosphamide (40HP) and doxorubicin, commonly administered to pre-pubertal candidates undergoing anti-cancer therapy, and the positive effects of EPA on these gonado-toxic compromised SCs.

## Materials and Methods

### Primary Cultures of Porcine Pre-Pubertal SCs

Animal studies were performed in agreement with the guidelines adopted by the Italian Approved Animal Welfare Assurance (A-3143-01) and European Communities Council Directive of 24 November 1986 (86/609/EEC). The experimental protocols were approved by the University of Perugia. Three large white neonatal pigs (15 to 20 days old) were used as SCs donors. Pure porcine neonatal SCs were isolated, purified and characterized according to previously established methods (47, 48).

### Chemotherapeutic Drugs/EPA Treatment

Pure porcine pre-pubertal SCs cultures were maintained at 37°C in a 5% CO2 humidified atmosphere in HAMF12 (Euroclone, Milan, Italy) supplemented with 0.166 nM retinoic acid (Sigma-Aldrich Co., St. Louis, MO, USA) and 5 mL/500 mL of Insulin-Transferrin-Selenium (ITS) + Premix (Cat. No. 354352; Corning, MA, USA) in the absence (untreated-control group) or presence of chemotherapeutic agents whose concentrations were chosen to include the range of detected serum levels in patients (49) and, cisplatin at the highest concentration plus EPA and 5-aza-2’-deoxy-cytidine (50, 51).

EPA effects were evaluated using the final concentration of 100 µM according to Finstad et al. (50).Variable effects of PUFA (polyunsaturated fatty acids) on proliferation, differentiation and apoptosis in leukemia cells have been reported in relation to cell line and fatty acid concentration. EPA; 20:5, n-3 turned out to be the most potent inhibitor of proliferation in a dose-dependent way; [100 µM] is the optimal concentration to get the maximum effect without inducing cytotoxicity.

The treatments were performed for 48 plus 24 h of recovery as follows:

- cisplatin 0.33 µM, 1.66 µM and 3.33 µM;
- 40HP 50 and 100 µM;
- doxorubicin 0.1 µM, 0.2 µM and 1 µM;
- cisplatin 3.33 µM plus EPA 100 µM;
- cisplatin 3.33 µM plus 5-aza-2’-deoxy-cytidine (5 AZA) 1 µM.

### Immunofluorescence Analysis

To detect the presence of 5hmC, immunostaining was performed according to previously reported methods with minor changes (52). Briefly, untreated and cisplatin plus EPA treated SCs monolayers were grown on glass chamber slides (LabTek II, Nunc; Thermo Fisher, Rochester, NY, USA) and fixed in 4% PFA-PBS for 30 min. Ten fixed cells then were subjected to permeabilization (PBS, 0.2% Triton X-100) for 5 min at room temperature and blocked with 0.5% BSA (Sigma-Aldrich) in PBS for 10 min prior to exposure to mouse 5hmC HMC/4D9 (EPIGENTEK, Farmingdale, New York, USA, 1:300) over night at +4°C. The cells were then washed in PBS three times for 5 min and then exposed to the secondary Alexa Fluor 488-labeled anti-mouse secondary antibody (Thermo Fisher Scientific, Waltham, MA, USA, 1:100). Thereafter, the cells were treated with RNAse (10 mg/ml; Sigma-Aldrich) and counterstained for 1 min with 4′,6-Diamidino-2-phenylindole dihydrochloride (DAPI) Sigma-Aldrich).

Negative controls bypassed the primary antibody treatment. Cells were mounted on slides with ProLong Gold anti-fade reagent (Molecular Probes). To evaluate the percentage of 5hmC positive cells, chamber slides were analyzed using a BX-41 microscope (Olympus, Tokyo, Japan) equipped with a fluorescence photocamera (F-viewer; Olympus); images were processed with Cell F imaging software (Olympus), and 10 different sections, containing at least 500 cells in total, were counted.

### Quantitative, Real-Time PCR

Total RNA was extracted using the TRIzol reagent (Invitrogen, Carlsbad, CA, USA), according to the manufacturer’s guidelines (53). Total RNA (1μg) was subjected to reverse transcription using Quanti Tect Reverse Transcription Kit (Qiagen, Hilden, Germany) in a final volume of 20 μL. Real-time PCR was performed using 16 ng of cDNA prepared by the RT reaction and SYBR Green master mix (Stratagene, Amsterdam, the Netherlands). The primer sequences of each gene are listed in **Table 1**. Real-time PCR was carried out in an Mx3000P cycler (Stratagene, Amsterdam, Netherlands) using FAM for detection and ROX as a reference dye. The mRNA level of each sample was normalized by β-actin mRNA and expressed as fold changes *vs* the level of the control group.

**Table 1:** Primer sequences for PCR analyses.

Gene	Forward sequences (5′–3′)	Reverse sequences (5′–3′)
AMH	GCGAACTTAGCGTGGACCTG	CTTGGCAGTTGTTGGCTTGATATG
Inhibin B	CCGTGTGGAAGGATGAGG	TGGCTGGAGTGACTGGAT
GDNF	TCAAGCCACCATCAGAAGA	TAGCCCAAACCCAAGTCA
β-actin	ATGGTGGGTATGGGTCAGAA	CTTCTCCATGTCGTCCCAGT

### Protein Extraction and Western Blot Analysis

Protein samples (70 µg) from total cell lysates were subjected to SDS-PAGE, electroblotted onto a nitrocellulose membrane (Schleicher and Schuell, Keene, NH, USA), and probed with anti GDNF EPR2714N (ab176564, abcam, Cambridge, UK) antibody. Immunoreactive bands were visualized using the ECL assay (Amersham Pharmacia Biotech, Little Chalfont, United Kingdom). Anti-b-Tubulin antibody (Millipore Sigma) was used to normalize. Images were acquired using the VersaDoc Imaging System (Bio-Rad, Hercules, CA, USA), and signals were quantified using Quantity One software (Bio-Rad).

### AMH and Inhibin B Secretion Assay

Aliquots of culture media from untreated and cisplatin plus EPA treated SCs were collected and stored at -20°C for subsequent assessment of AMH (AMH Gen II ELISA, Beckman Coulter, Webster, TX, USA) and inhibin B (inhibin B Gen II ELISA, Beckman Coulter) secretion levels as previously described (54).

### Chemicals and Reagent

Ketamine (Ketavet 100) was purchased by Intervet, Milan, Italy.

Dexmedetomidine (Dexdomitor) was purchased by Orion Corporation, Finland.

HAMF12, PBS, HBSS were purchased by Euroclone, Milan, Italy.

Retinoic acid, PFA, bovine serum albumin (BSA) fraction V (fatty acid free), Triton X-100, RNase, 4′,6-Diamidino-2-phenylindole dihydrochloride (DAPI) were purchased by Sigma-Aldrich Co., St. Louis, MO, U.S.A.

Insulin-Transferrin-Selenium (ITS) + Premix was purchased by Corning, MA, U.S.A.

Cisplatin and Doxorubicin were purchased by TEVA, Milan, Italy.

4-hydroxxycyclosphosmamide (4OHP) was purchased by Niomech, Bielefeld, Germany.

Eicosapentaenoic acid (EPA, 20:5, n-3), anti-β-Tubulin antibody and 5-aza-2’-deoxycytidine (5-aza) were purchased from MilliporeSigma, Burlington, MA, U.S.A.

Glass chamber slides (LabTek II), were purchased by Nunc, Thermo Fisher, Rochester, NY, U.S.A.

5-hydroxymethylcytosine (5-hmC), HMC/4D9 was purchased by EPIGENTEK, Farmingdale, New York, U.S.A.

Secondary Alexa Fluor 488-labeled anti-mouse secondary antibody was purchased by Thermo Fisher Scientific, Waltham, MA, U.S.A.

ProLong Gold anti-fade reagent was purchased by Molecular Probes, Eugene, Oregon, U.S.A.

BX-41 microscope, fluorescence photocamera (F-viewer) and Cell F imaging software were purchased by Olympus, Tokyo, Japan.

TRIzol reagent was purchased by Invitrogen, Carlsbad, CA, U.S.A.

Quanti Tect Reverse Transcription Kit was purchased by Qiagen, Hilden, Germany.

SYBR Green master mix was purchased by Stratagene, Amsterdam, the Netherlands).

Mx3000P cycler was purchased by Stratagene, Amsterdam, Netherlands.

Nitrocellulose membranes were purchased by Schleicher and Schuell, Keene, NH, U.S.A.

anti GDNF EPR2714N (ab176564) was purchased by Abcam, Cambridge, UK.

ECL assay was purchased by Amersham Pharmacia Biotech, Little Chalfont, United Kingdom.

VersaDoc Imaging System and Quantity One software were purchased by Bio-Rad, Hercules, CA, USA.

AMH (AMH Gen II ELISA) and inhibin B (Inhibin B Gen II ELISA) were purchased by Beckman Coulter, Webster, TX, U.S.A.

SigmaStat 4.0 software was purchased by Systat Software Inc., CA, U.S.A.

### Statistical Analysis

Values are reported as the means ± S.E.M. of three independent experiments, each performed in triplicate. Statistical analysis was performed using the paired Student’s t-test with SigmaStat 4.0 software (Systat Software Inc., CA, USA). All tests were performed in triplicate, and differences were considered statistically significant at p <.05, p <.01, and p <.001 compared to untreated SCs.

## Results

### SCs Purification and Characterization

SCs isolated from testes of Large White pre-pubertal pigs were highly purified (95%) as indicated by the immunostaining for AMH, a specific and unique pre-pubertal SCs marker.

The presence of contaminating cells was extremely low (<5%) according to a previously describe staining technique by Arato et al. (54).

### 5hmC Immunofluorescence Analysis

**Figure 1:**
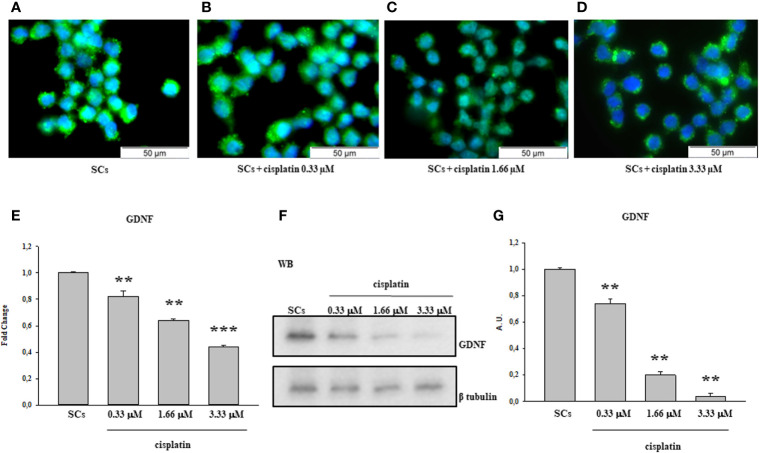
Cisplatin treatment: Immunofluorescence Analysis - Real-Time PCR -WB and densitometric analysis. 5hmC was evaluated by IF **(A–D)** and GDNF by Real Time PCR **(E)** WB and densitometric analysis **(F, G)** in control group and after cisplatin 0.333, 1.66 and 3.33µM treatment. See text for more details. Data represent the mean ± S.E.M. (**p < 0.01 and ***p < 0.001 respect to untreated SCs) of three independent experiments, each performed in triplicate.

After 48 h of treatment to various concentrations of Cisplatin, including 0.33 µM, 1.66 µM and 3.33 µM immunofluorescence analysis showed a statistical reduction of 5hmC in a dose-dependent manner compared to untreated SCs (**Figures 1A–D**).

**Figure 2:**
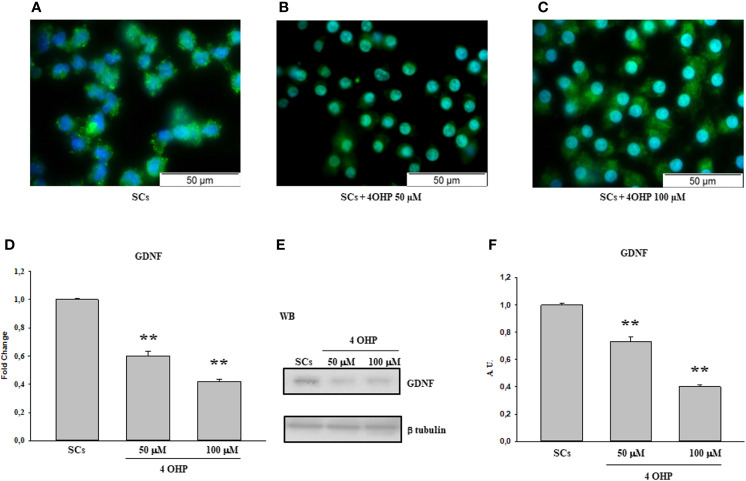
4OHP treatment: Immunofluorescence Analysis - Real-Time PCR – WB and densitometric analysis. 5hmC was evaluated by IF **(A–C)** and GDNF by Real Time PCR **(D)** WB and densitometric analysis **(E, F)** in control group and after 40HP 50 and 100 µM treatment. See text for more details. Data represent the mean ± S.E.M. (**p < 0.01 respect to untreated SCs) of three independent experiments, each performed in triplicate.

**Figure 3:**
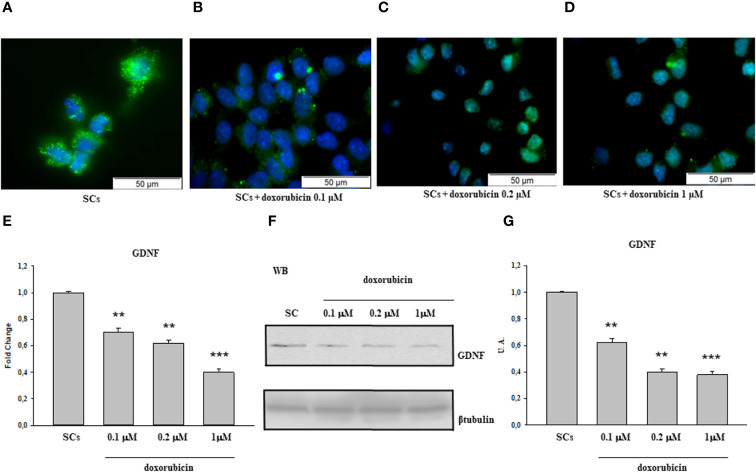
Doxorubicin treatment: Immunofluorescence Analysis - Real-Time PCR - WB and densitometric analysis. 5hmC was evaluated by IF **(A–D)** and GDNF by Real Time PCR **(E)** WB and densitometric analysis **(F, G)** in control group and after doxorubicin 0.l, 0.2 and 1 µM treatment. See text for more details. Data represent the mean ± S.E.M. (**p < 0.01 and ***p < 0.001 respect to untreated SCs) of three independent experiments, each performed in triplicate.

The same behavior was observed following treatment with 50 and 100 40HP (**Figures 2A–C**) and at the 0.1 µM, 0.2 µM and 1µM doxorubicin concentrations in comparison with the control group (**Figures 3A–D**).

### GDNF Protein and Gene Expression

The gene expression levels of GDNF were significantly down-regulated after treatment to all three cisplatin concentrations in a dose-dependent manner compared to untreated SCs.

Similarly, the protein expression level of GDNF also was significantly down-regulated in all experimental conditions compared to the untreated SCs (**Figures 1E–G**).

A similar trend was recorded upon 50 and 100 of 40HP, in term of gene and protein expression levels, in comparison with the control group (**Figures 2D–F**). In addition, compared with the control group, the gene and protein expression levels of GDNF were significantly down-regulated also following treatment to all three doxorubicin concentrations in a dose-dependent manner compared to untreated SCs (**Figures 3E–G**).

**Figure 4:**
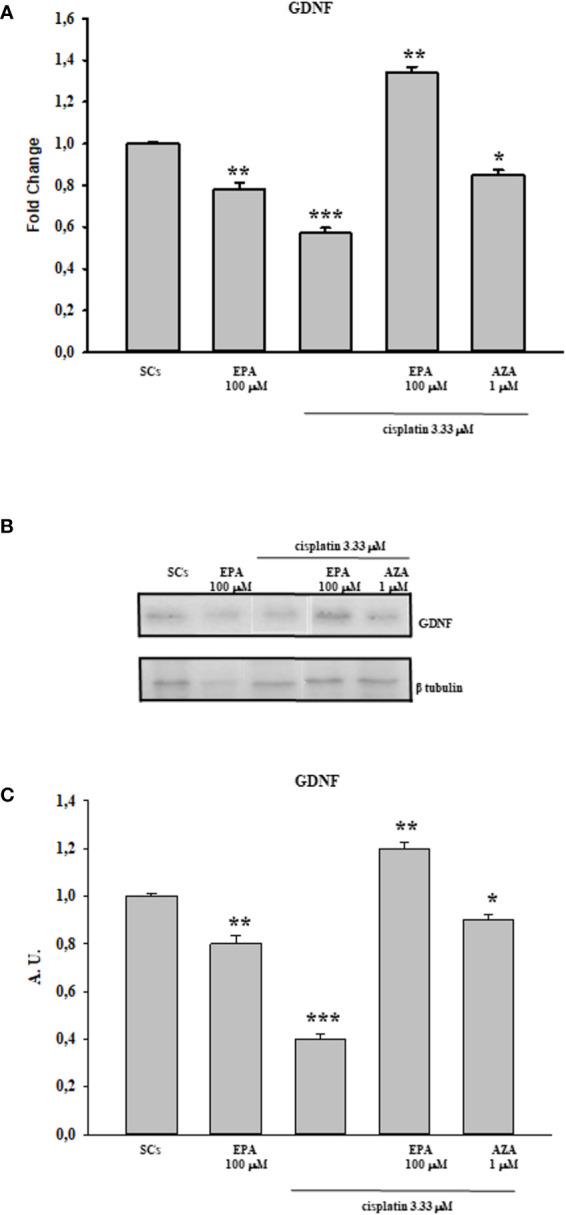
Cisplatin plus EPA treatment: Real-Time PCR - WB and densitometric analysis. Evaluation of the protective effect of EPA 100µM on GDNF gene **(A)** and protein expression upon cisplatin 3.33 µM treatment by WB **(B)** and densitometric analysis **(C)**. See text for more details. Data represent the mean ± S.E.M. (*p < 0.05, **p < 0.01, and ***p < 0.001 respect to untreated SCs) of three independent experiments, each performed in triplicate.

Finally, the **Figures 4A–C** shows the protective effect of EPA 100 µM by an up-regulation in both gene and protein expression levels of GDNF at the 3.33 µM cisplatin concentration in comparison with the control group. SCs were also treated with the combination of cisplatin 3.33 µM plus 5AZA 1µM in order to further validate the involvement of the methylation process in GDNF gene regulation; this molecule is a well-known demethylating agent used in various therapeutic anticancer treatments. The figure shows that the effect induced by 5AZA with cisplatin treatment is comparable to that caused by EPA with cisplatin treatment, thus assuming a link between the protective effect of the fatty acid and a demethylating action.

### Sertoli Cell Functional Competence

**Figure 5:**
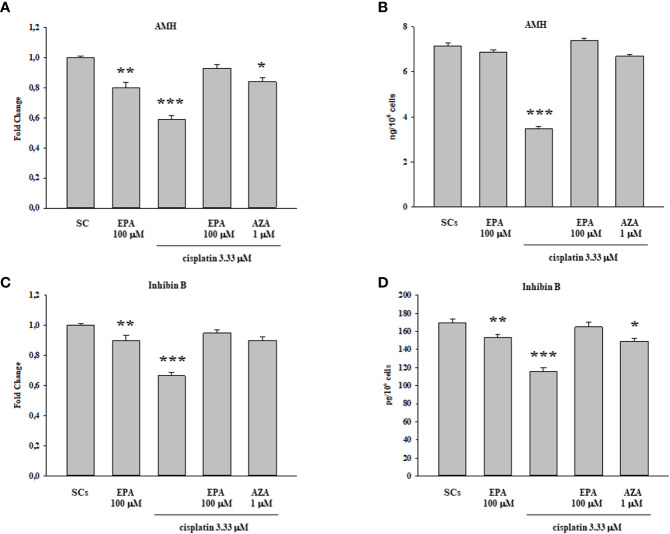
Cisplatin plus EPA treatment: SCs functional analysis. AMH **(A, B)** and inhibin B **(C, D)** were evaluated by Real Time PCR **(A, C)** and ELISA assay **(B, D)** following cisplatin 3.33µM plus EPA 100µM treatment. Data represent the mean ± S.E.M. (*p < 0.05, **p < 0.01, and ***p < 0.001 respect to untreated SCs) of three independent experiments, each performed in triplicate.

The gene expression levels of AMH and inhibin B, as specific markers of SCs function, were significantly down-regulated following cisplatin 3.33 µM treatment but, were significantly recovered only when coupling the cisplatin 3.33 µM and EPA 100 µM treatment, with values comparable to the control group (**Figures 5A, C**). Similarly the secretion of AMH and inhibin B, was significantly decreased after cisplatin 3.33 µM treatment but were significantly recovered following treatment with cisplatin 3.33 µM plus EPA 100 µM with values comparable to the control group (**Figures 5B, D**).

## Discussion

The increasing numbers of male pre-pubertal cancer survivors has spurred interest regarding the quality of adult life in these individuals, with increasing importance given to reducing the well-defined long-term side effects of toxic agents used in anti-cancer therapies (55, 56). At present, chemotherapeutic drugs are considered to be highly toxic to the pre-pubertal testis by direct DNA and RNA damage and the activation of apoptotic pathways (57).

Clearly the use of these agents pose a significant likelihood of reducing the establishment of a normal adult fertility potential in young boys undergoing cancer treatment (58, 59) making, therefore, fertility preservation, as determined by intra-testicular cellular events in pre-pubertal testes, most important. In the present study we addressed the effect of EPA on pre-pubertal porcine SCs “*in vitro*” which had been previously exposed to a clinically-relevant range of the anti-cancer agents cisplatin (0.33, 1.66 and 3.33 µM), 40HP (50 and 100 µM) and doxorubicin, (0.1, 0.2 and 1 µM) all of which have deleterious effects on normal male fertility.

Determining the impact of these gonado-toxic anti-cancer agents on the compromised pre-pubertal porcine SCs was considered appropriate because animal studies have determined that anti-cancer treatment, in the form of cytotoxic chemotherapy and radiotherapy, can cause long-lasting damage to SCs critical in promoting germ cell survival (49, 60). Establishment of complete spermatogenesis will depend on the degree of damage caused to GSC, either directly or indirectly following impairment of SCs in the pre-puberal testis; and, of course, pre-pubertal boys subjected to gonado-toxic cancer treatment, as previously described, can likely suffer significant depletion of GSC due to SC damage and ultimately resulting in permanent azoospermia (8). GSC is the only germ cell population present during pre-pubertal life until the onset of spermatogenesis at puberty at which time they divide and ultimately enter meiosis to form spermatozoa in the adult (61–63).

SCs are a critical component of the GSC niche where homeostasis is maintained by the interplay of several signaling pathways and growth factors such as the GDNF (64). The GDNF, interacting with the RET/GFRA1 receptor complex on the surface of undifferentiated spermatogonia, acts as a mitogenic agent regulating the renewal of the pool of GSC spermatogonial stem cells, a target of gonado-toxic therapies (28). In particular, RET and GFRA1 are concomitantly expressed in A_paired_ and some A_aligned_ GSC. The role of GDNF signaling in GSC proliferation and differentiation has been unequivocally demonstrated by studies *in vivo* using Gdnf+/- mice and mice testis as well as by studies “*in vitro”* in which GDNF was identified as an essential factor for spermatogonial stem cell (65). Additionally, by using xenograft transplantations of neonatal knockout testes, Naughton et al. demonstrated that the absence of GDNF or its receptors RET and GFRA1 after birth led to a lack of GSC and failure of spermatogenesis (29).

In the current study, we observed that there was a significant reduction in both gene and protein expression levels of GDNF after treatment with all three cisplatin concentrations in a dose-dependent manner compared to untreated SCs. A similar trend, in a dose-dependent manner in comparison with the control group, was recorded following treatment with 50 and 100 µM 40HP and also following treatments with all three doxorubicin concentrations. These effects were concomitant with a significant reduction of 5hmC levels on the entire genomic DNA in a dose-dependent manner following treatment with all three chemotherapeutic drugs when compared to control groups. After observing the damage of chemotherapy treatments on porcine SCs culture, in terms of gene and protein expression of GDNF and the reduction of 5hmC levels, we have shown the protective effect of EPA only at the highest concentration of cisplatin of 3.33µM, since this drug had a greater toxic effect than exposure to 4OHP or doxorubicin.

After observing that EPA treatment resulted in maintaining the functional status of SCs by increasing both gene and protein expression levels of GDNF, compared with the control group, we hypothesize the involvement of a demetilating action in up-regulating GDNF gene transcription. In support of this speculation, SCs were treated with cisplatin plus 5AZA (a well-known demethylating agent used in different chemotherapy treatments of tumor pathologies) obtaining an up-regulation of GDNF expression comparable to that induced by EPA treatment, even though the treatment with fatty acid showed a greater efficacy. The use of EPA as a demethylating agent avoids the onset of the toxic effects that normally accompany the chemotherapy treatment with generalized-acting demethylating agents such as 5-AZA. EPA in fact, acts to recover a correct gene expression if this is modified (66).

In the present study, we further assessed the effect of EPA on SCs functional competence the evaluation of AMH and inhibin B secretion, two well-known specific and important markers of SCs functionality (67, 68). We observed an up-regulation in both AMH gene expression and secretion following cisplatin 3.33 µM plus EPA 100µM treatment with values comparable to the control group. This is consistent with previous findings that AMH represents one of the most useful markers of testis functionality during the pre-pubertal period (54). In addition, we observed an increase in inhibin B gene expression and secretion upon cisplatin 3.33 µM plus EPA 100µM treatment also with values comparable to the control group. Inhibin B measurements are used in clinical practice to evaluate the presence and function of SCs during childhood and may be a promising indicator of diminished sperm production as a result of cytotoxic chemotherapy (69).

The present work, pertinent to male infertility in pre-pubertal candidates for gonado-toxic therapies, suggests that EPA acts as a modulator of SCs GDNF, promoting its epigenetic regulation and restoring SCs function damaged following radio or chemotherapy drugs.

Because EPA resulted in the recovery of GDNF in SCs “*in vitro”*, a mechanism by which new germ cells are induced “*in vivo”*, we hypothesize that the use of EPA may be useful in suppressing the deleterious effects of chemo and or radiotherapy on fertility potential for cancer in pre-pubertal and pubertal boys. In conclusion, with the growing population of early childhood cancer survivors, there is an urgent need to develop new strategies against oncological treatments to safeguard the fertility potential of this group of patients, strategies such as suggested by the results of this study.

## Data Availability Statement

The raw data supporting the conclusions of this article will be made available by the authors, without undue reservation.

## Ethics Statement

The animal study was reviewed and approved by Italian Approved Animal Welfare Assurance (A-3143-01).

## Author Contributions

All authors contributed to the article and approved the submitted version. IA, VC, and FM designed and drafted the manuscript. The experimental procedures and data analysis were performed by CB, CL, PF, KP, AD’A, MA, MC, and TB. RC and DC gave experimental guidance. AV and GL supervised and revised the manuscript. All authors contributed to the article and approved the submitted version.

## Funding

This work was part of the project ‘Effetti acuti di agenti chemioterapici sulla regolazione di cellule del Sertoli di suino prepubere’ supported by a grant to TB from University of Perugia (Fondo Ricerca di Base dell’Università degli Studi di Perugia, RB2019TBAR).

## Conflict of Interest

The authors declare that the research was conducted in the absence of any commercial or financial relationships that could be construed as a potential conflict of interest.
